# Functional Larynx Preservation in Patients With Locally Advanced Squamous Cell Carcinoma of the Larynx and Hypopharynx Treated With Induction Chemotherapy vs. Concurrent Chemoradiation Alone

**DOI:** 10.7759/cureus.16310

**Published:** 2021-07-11

**Authors:** Anthony D Nehlsen, Eric J Lehrer, Daniel R Dickstein, Marshall R Posner, Krzysztof Misiukiewicz, Jerry Liu, Vishal Gupta, Richard L Bakst, Sonam Sharma

**Affiliations:** 1 Radiation Oncology, Mount Sinai, New York, USA; 2 Radiation Oncology, Icahn School of Medicine at Mount Sinai, New York City, USA; 3 Radiation Oncology, Icahn School of Medicine at Mount Sinai, New York, USA; 4 Medical Oncology, Icahn School of Medicine at Mount Sinai, New York, USA

**Keywords:** larynx preservation, hypopharynx, radiation, squamous cell carcinoma, induction chemotherapy

## Abstract

Objectives

Chemoradiation therapy (CRT) has been established as a standard treatment for locally advanced hypopharynx/larynx squamous cell carcinoma (SCC) but the role of induction chemotherapy (IC) remains unclear. The primary outcome of this study is to determine whether functional larynx-preservation survival (FLPS) is improved with the addition of IC in these patients. Secondary outcomes were overall survival (OS), progression-free survival (PFS), distant metastasis-free survival (DMFS), and laryngectomy rates.

Methods

Records for patients with AJCC 8th edition clinical stage III-IVB laryngeal and hypopharyngeal SCC treated with CRT +/- IC from 2005-2019 were reviewed. FLPS was defined as time until death, progression, laryngectomy, or non-functional larynx. Kaplan-Meier curves were generated for FLPS, OS, PFS, and DMFS. Outcomes were compared using the stratified log-rank test. Laryngectomy rates were compared using Fisher’s exact test.

Results

We included 52 patients with laryngeal and 38 with hypopharyngeal SCC (n=90); 19 patients with laryngeal SCC and 19 with hypopharyngeal SCC received IC (median three cycles). There were no differences in the three-year FLPS (61% vs 67.8%; p=0.88), OS (73.9% vs 86.2%; p=0.42), PFS (53.6% vs 62.6%; p=0.44), or DMFS (65.2% vs 71.5%, p= 0.85) between patients who did and did not receive IC all patients. Laryngectomy rates did not differ with and without IC (18.4 % vs 7.7%; p=0.19).

Conclusion

In this study of advanced laryngeal and hypopharyngeal SCC, IC did not improve three-year FLPS, OS, PFS, or laryngectomy rates compared to CRT alone. A large prospective series would provide a more robust understanding of the role of IC in this group of patients.

## Introduction

In addition to local control and survival outcomes, optimizing functional organ preservation in patients with locally advanced laryngeal and hypopharyngeal squamous cell carcinomas (SCC) continues to be an important outcome measure due to the improved quality of life measures associated with larynx preservation [1]. Laryngectomy is associated with significant morbidity, including the development of difficulties with speech and swallowing, and offers no benefit in terms of overall survival (OS) compared to concurrent chemoradiation therapy (CRT) for many patients [2-4]. It is important to note, however, that laryngectomy (+/- adjuvant therapy) can improve local control and possibly OS in patients with T4a primary lesions [5, 6]. In patients without T4a disease, high rates of local control and larynx preservation are possible with the use of CRT, and those who do experience local failures can often be effectively salvaged with surgery [7]. Numerous clinical trials have analyzed the role of CRT to improve larynx preservation rates, with both induction chemotherapy (IC) followed by radiation and CRT being used. 

The Veteran Affairs Larynx Trial established the role of combined modality therapy in the treatment of locally advanced laryngeal cancer, showing equivalent OS and a 64% laryngeal preservation rate at two years in patients receiving IC followed by radiation therapy (RT) compared to those receiving laryngectomy with postoperative RT [2]. This was further examined in hypopharyngeal SCC by EORTC trial 24891, which also demonstrated a 22% rate of survival with an intact larynx at five years and similar rates of local failure, OS, and progression-free survival (PFS) in the IC plus RT arm compared to the group receiving up-front surgery [8]. Intensified IC in SCC of the larynx and hypopharynx using docetaxel, cisplatin, and 5-FU (TPF) compared to cisplatin and 5-FU (PF) has also been studied, with randomized data demonstrating improvements in larynx preservation rates and OS [9, 10]. 

Definitive CRT has also been studied in the randomized setting, with RTOG 91-11 showing improved local control and larynx preservation at 10 years compared to sequential chemotherapy and RT or RT alone, with similar rates of OS [3]. Additionally, combining CRT and induction therapy has emerged as a treatment option. The TREMPLIN trial, which randomized patients with stage III-IV laryngeal or hypopharyngeal SCC to CRT with cisplatin or cetuximab after induction with three cycles of TPF resulted in excellent rates of larynx preservation (87% vs 82%, respectively) [11].

Despite the improvement in outcomes seen in the trials above, conflicting studies have not shown a significant benefit to induction chemotherapy when compared to CRT alone. For example, the PARADIGM and DeCIDE trials, each of which included a significant number of patients with SCC of the larynx and hypopharynx, showed no benefit to the use of IC when compared to CRT alone [12, 13]. Similarly, the MACH-NC meta-analysis found improved outcomes with CRT when compared to IC followed by RT [14].

This data, in combination with the significantly higher rates of treatment-related toxicity and complexity seen in patients undergoing IC unless treated in experienced multi-disciplinary centers, makes the routine use of IC in the treatment of SCC of the larynx and hypopharynx a topic of ongoing discovery [9-13]. In this study, we aim to compare the rates of functional-larynx preservation survival in patients with stage III-IV SCC of the larynx and hypopharynx treated with IC followed by CRT to those patients receiving CRT alone using a retrospective cohort of patients treated at a large academic medical center.

## Materials and methods

This study was approved by the Mount Sinai Institutional Review Board. We identified the records of patients with locally advanced clinically staged III-IV (AJCC 8th edition) SCC of the larynx and hypopharynx treated at our institution from 2005 through 2018. Eligible patients were at least 18 years of age, had pathologic confirmation of SCC of the larynx or hypopharynx, with localized disease at presentation, and were treated with multi-agent IC (TPF or PF) followed by definitive CRT or with definitive CRT alone. Charts were reviewed for radiation, chemotherapy, staging, pathologic, demographic, and outcomes data. 

The aim of this study was to compare treatment outcomes in patients treated with IC followed by definitive CRT to patients treated with CRT alone when using laryngeal preservation for locally advanced larynx and hypopharynx SCC. The primary endpoint was functional larynx-preservation survival (FLPS), which was defined as the time until death, local progression, laryngectomy, or non-functional larynx. Secondary endpoints were OS, PFS, and rates of laryngectomy at the last follow-up. Response to IC was measured using computed tomography or positron emission tomography (PET) imaging based on the studies available.

Statistical analyses were conducted using R Studio ver 1.1.419 (R Studio, Boston, MA). The ‘ggplot2’ package ver 3.2.1 was used to generate Kaplan-Meier curves and log-rank tests. The ‘survival’ package ver 3.1-8 was used to conduction the cox proportional hazards models. Each time-to-event outcome (OS, PFS, and FLPS) was stratified by receipt of induction chemotherapy and then by disease subsite (larynx and hypopharynx). The stratified log-rank test was used to compare these outcomes by the aforementioned groupings, where the null hypothesis was rejected for p<0.05. Univariate and multivariate cox proportional hazards models were used to assess the impact of covariates on OS, PFS, and FLPS. We included T/N stage, smoking status, and receipt of induction chemotherapy in the multivariate analysis due to the clinical behavior of SCC of the larynx and hypopharynx. We included any covariates with a p-value of 0.3 on the univariate analysis in the multivariate analysis. Goodness of fit of the multivariate model was assessed using the log-rank test, where a backward selection method was used to determine the optimal multivariate model. Satisfaction of the proportional hazards assumption was assessed using Schoenfeld’s Test and visual inspection of Schoenfeld Residuals.

## Results

A total of 90 patients meeting our inclusion criteria were identified: 52 of these patients had SCC of the larynx, of which 19 received IC and 33 were treated with CRT alone. Additionally, 38 patients had SCC of the hypopharynx, of which 19 received IC, and 19 were treated with CRT alone. The median follow-up for the cohort was 24 months (range 2-121). Patient characteristics are further detailed in Table 1. As shown in Table 2, the two cohorts were well matched for T stage, smoking history, primary site of disease, ECOG performance status, and age. However, there were significantly more patients with N2 or N3 disease (p=0.009) and clinical stage IV disease (p=0.0001) in the IC chemotherapy group.

**Table 1:** Patient Demographics

	Induction (n=38)	Non-Induction (n=52)
Age (median)	63	66
Gender		
Male	28 (73.7%)	45 (86.5%)
Female	10 (26.3%)	7 (13.5%)
Ethnicity		
White	25 (65.8%)	23 (44.2%)
African American	8 (21.1%)	13 (25.0%)
Other	5 (13.1%)	16 (30.8%)
ECOG		
0	6 (18.2%)	13 (26.0)
1	24 (72.7%)	26 (52.0%)
≥2	3 (9.1%)	11 (22.0%)
Smoking history		
Yes	31 (81.6%)	44 (84.6%)
No	7 (18.4%)	8 (15.4%)

**Table 2:** Tumor Characteristics

	Induction (n=38)	Non-Induction (n=52)	p-Value
Primary site			
Larynx	19 (50.0%)	33 (63.5%)	0.28
Hypopharynx	19 (50.0%)	19 (36.5%)	
T Stage			
T1-3	25 (69.4%)	43 (82.7%)	0.20
T4	11 (30.6%)	9 (17.3%)	
N Stage			
N0-1	12 (33.3%)	33 (63.5%)	0.009
N2-3	24 (66.7%)	19 (36.5%)	
AJCC Clinical Stage			
III	5 (13.2%)	27 (51.9%)	0.0001
IV	33 (86.8%)	25 (48.1%)	

The median radiation dose received was 70 Gy (range 60-75) in 35 fractions (range 30-50). A median of three cycles (range 1-4) of chemotherapy were given in those receiving IC. Twenty-seven patients were treated with TPF and 11 patients were treated with PF.

**Figure 1:**
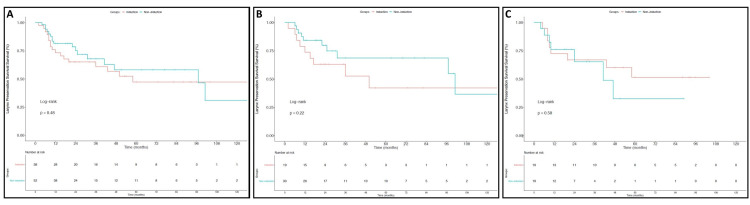
Functional Larynx Preservation Survival This figure depicts functional larynx preservation survival (FLPS) outcomes for all patients (A), larynx SCC patients (B), and hypopharynx SCC patients (C). Outcomes for patients who did not receive induction chemotherapy are in blue and those who did receive induction chemotherapy are in red.

FLPS rates at three years did not differ significantly between the patients receiving IC and those receiving CRT alone for the entire cohort (Figure 1A), with rates of 61% and 67.8% respectively (p=0.48). Similar findings were seen on subset analysis by primary tumor site for those with SCC of the larynx (52.6% vs 68.8%; p=0.22) and hypopharynx (66.9% vs 65.3%; p=0.58). These findings are shown in Figure 1B and Figure 1C, respectively. 

**Figure 2:**
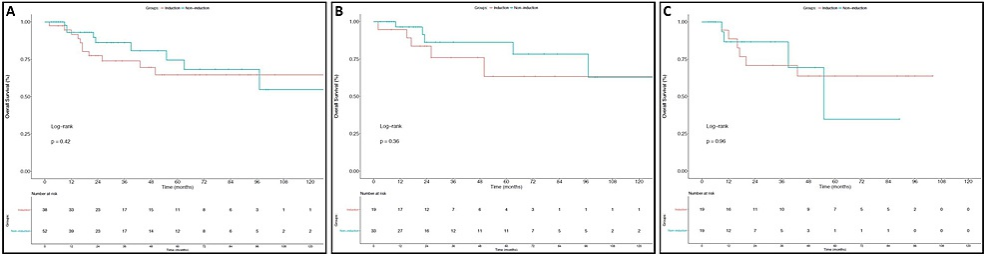
Overall Survival This figure depicts overall survival (OS) outcomes for all patients (A), larynx SCC patients (B), and hypopharynx SCC patients (C). Outcomes for patients who did not receive induction chemotherapy are in blue and those who did receive induction chemotherapy are in red.

**Figure 3:**
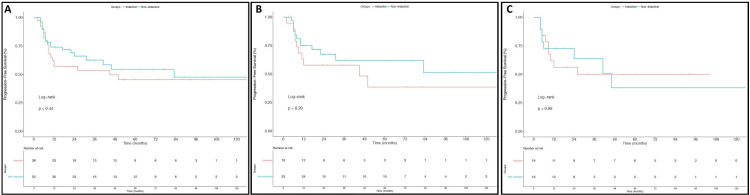
Progression-Free Survival This figure depicts progression-free survival (PFS) outcomes for all patients (A), larynx SCC patients (B), and hypopharynx SCC patients (C). Outcomes for patients who did not receive induction chemotherapy are in blue and those who did receive induction chemotherapy are in red.

**Figure 4:**
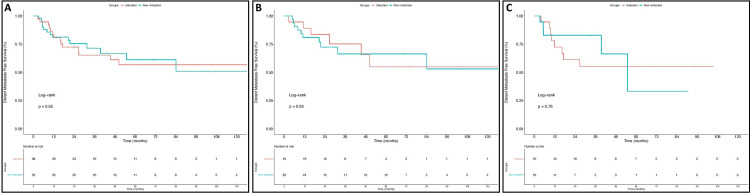
Distant Metastasis-Free Survival This figure depicts distant metastasis-free survival (DMFS) outcomes for all patients (A), larynx SCC patients (B), and hypopharynx SCC patients (C). Outcomes for patients who did not receive induction chemotherapy are in blue and those who did receive induction chemotherapy are in red.

Rates of three-year OS were also similar in the entire cohort for patients treated with IC and those treated with CRT alone (Figure 2A) (73.9% vs 86.2%; p=0.42). As shown in Figures 2B-2C respectively, there were also no significant differences observed in three-year OS for larynx (76% vs 86.3%; p=0.36) or hypopharynx patients (70.8% vs 86.7%; p=0.96). Additionally, PFS at three years did not differ between patients who did and did not receive IC for the entire cohort (Figure 3A) (53.6% vs 62.6%; p=0.44) or on subset analysis for patients with SCC of the larynx (Figure 3B) (57.9% vs 61.8%; p=0.29) or hypopharynx (Figure 3C) (50% vs 63.6%; p=0.99). Finally, as shown in Figure 4A, three-year DMFS rates for all patients did not differ between those who did and did not receive IC (65.2% vs 71.5%, p=0.85). As depicted in Figures 4B-4C, this was also true for patients with laryngeal SCC (75.2% vs 66.3%, p=0.93) and hypopharyngeal SCC (55.2% vs 82.9%, p=0.76). 

Laryngectomy rates are shown in Table 3. In the entire cohort, 11 patients (12.2%) required laryngectomy, with seven (18.4%) occurring in the IC group compared to 3 (7.7%) in the patients treated with CRT alone (p=0.19). In the larynx and hypopharynx subgroups, these rates were 21.1% vs 6.1% (p=0.18) and 15.8% vs 10.5% (p>0.99), respectively. Laryngectomy was performed for recurrence in 5/7 (71%) patients in the IC group (with the other two being for a non-functional larynx) and in all four patients in the CRT alone group.

**Table 3:** Salvage Laryngectomy Rates

	Induction (n=38)	Non-Induction (n=52)	p-Value
Entire cohort	7 (18.4%)	4 (7.7%)	0.19
Larynx	4 (21.1%)	1 (6.1%)	0.18
Hypopharynx	3 (15.8%)	3 (10.5%)	>0.99

On multivariate analysis (MVA) (Table 4 for FLPS), induction chemotherapy was not associated with improved FLPS, OS, or PFS (p>0.05). N2 disease was associated with worse outcomes for FLPS (HR 8.56, 95% CI 1.63-45.01, p=0.01), OS (HR: 3.57, 95% CI: 1.22-10.45, p=0.02), PFS (HR 3.76, 95% CI 1.44-9.77, p=0.007), and DMFS (HR: 16.32, 95% CI: 1.20-222.26, p=0.04). N3 disease was also associated with worse outcomes for FLPS (HR 41.4, 95% CI 5.19-330.34, p<0.001), OS (HR 18.35, 95% CI 2.13-157.73, p=0.008), PFS (HR 24.8, 95% CI 4.56-135.03, p<0.001), and DMFS (HR: 269.58, 95% CI: 11.46-6,341.03, p<0.001). There was no difference in three-year FLPS (63.6% vs 47.2%, p=0.93), OS (61% vs 81.6%, p=0.85), or PFS (33.5 vs 37.9%, p=0.48) in patients with N2-3 disease receiving IC compared to those who did not, respectively.

**Table 4:** Multivariate Analysis for FLPS FLPS: functional larynx-preservation survival

Variable	Hazard Ratio (HR)	95% CI	p-Value
Age	0.98	0.94-1.02	0.33
Induction			
Yes	Ref	Ref	Ref
No	0.88	0.32-2.46	0.81
Smoking			
No	Ref	Ref	Ref
Yes	3.3	0.31-34.70	0.32
T Stage			
1	Ref	Ref	Ref
2	0.34	0.03-4.50	0.41
3	0.99	0.09-11.37	0.99
4	1.11	0.10-12.81	0.94
N Stage			
0	Ref	Ref	Ref
1	2.66	0.40-17.75	0.31
2	8.56	1.63-45.01	0.01
3	41.4	5.19-330.34	<0.001

Response to IC was available in 25 patients, with one (4%) patient demonstrating progression of disease, 20 (80%) showing a partial response (PR), and four (16%) exhibiting a complete response (CR). For the larynx SCC subset, 11 patients demonstrated a PR and two exhibited a CR. One patient in the hypopharynx subset suffered from POD while nine experienced a PR and two experienced a CR.

## Discussion

In our study population, the use of IC did not improve FLPS, OS, PFS, or DMFS in patients treated with definitive CRT for locally advanced SCC of the larynx or hypopharynx. While patients in the IC group did have a higher proportion of patients with N2/N3 disease, which was associated with higher rates of failure, multivariate analysis did not demonstrate any benefit to IC in our cohort of patients. However, the imbalance of nodal disease burden between the two groups remains a confounding variable that could have influenced the results. Additionally, clinical trials, such as TREMPLIN and GORTEC 2000-01, demonstrated high rates of functional larynx preservation in patients with stage III/IV laryngeal or hypopharyngeal SCC receiving induction chemotherapy, suggesting that there may be a subset of patients who would significantly benefit from the use of IC prior to definitive CRT [10, 11].

Therefore, the implementation of strategies aimed at identifying those patients at the highest risk of local failure may help guide the use of IC. Due to the high propensity for local failure requiring laryngectomy seen in patients with T4 tumors in the VA Larynx Trial, T stage has since been viewed as a strong predictor for local failure after CRT [2]. However, in our analysis, T stage was not significantly linked to lower rates of FLPS, OS, PFS, or DMFS. Other studies have also found no link between T stage and local recurrence rate, finding instead that primary tumor volume may be a better predictive tool [15-18]. Our study showed a strong correlation between advanced nodal status (N2-3) and outcomes, which has been corroborated by a number of studies finding links between nodal stage and volume with survival and control outcomes [15, 18, 19]. Finally, there is additional data supporting the use of PET imaging, including metabolic tumor volume and maximum SUV, as a predictor for larynx preservation and OS [20, 21]. Although additional studies are needed to validate these findings, risk-stratifying patients based on primary tumor volume, the extent of nodal involvement, and PET findings may provide a better means of identifying those that may benefit from intensified therapies such as IC when being treated with larynx preservation therapy. 

Identifying patients who are likely to tolerate and respond to IC is an important avenue of future research. Evidence by Liu et al. suggests that patients with advanced SCC of the hypopharynx who completely respond to induction chemotherapy have excellent rates of larynx preservation and survival. However, those treated with larynx preservation who achieved a partial response or less did markedly worse in terms of both OS and larynx preservation [22]. While our cohort lacked the statistical power for a similar type of analysis, this may represent a method for the identification of patients who may benefit from more aggressive treatment options or surveillance schedules. Other studies of organ preservation protocols using induction chemotherapy in both laryngeal and hypopharyngeal SCC have also found improved survival and organ preservation outcomes in patients with significant responses to IC compared to those with partial response [23, 24]. This suggests that the selection of patients likely to have significant responses to chemotherapy may allow for the identification of patients with locally advanced disease that are most likely to achieve long-term survival with meaningful larynx preservation. One such strategy by Wichmann et al. used PET and endoscopic evaluation after one cycle of multi-agent induction chemotherapy to prospectively create a prognostic scoring system that predicted laryngectomy-free survival and OS in patients undergoing larynx preservation therapy. Their prognostic scoring also aided in the early recognition of non-responders who were more likely to benefit from early salvage laryngectomy [25]. While additional prospective trials are needed to validate these findings as a means of predicting which patients are most likely to respond to IC, the use of prognostic scoring systems provides an exciting area of future study. A large randomized trial (SALTORL), comparing definitive CRT to IC followed by RT in patients locally advanced larynx and hypopharynx SCC, is currently ongoing and will hopefully provide more definitive insight into the role of IC in this setting.

Our study has a number of important limitations. Notably, this was a retrospective analysis, which makes it difficult to account for confounding biases that may have affected our results and limits the ability to determine causation from our findings. Patients in the IC arm had a higher prevalence of stage IV and N2 disease, which could have affected larynx preservation or OS rates, particularly due to the worse prognosis associated with N2 disease. The groups were otherwise well balanced in terms of tumor characteristics. Additionally, the number of cycles and selection of chemotherapeutic agents given to each patient during their induction regimens was not uniform which may have affected disease outcomes.

## Conclusions

In this retrospective analysis of patients with stage III-IV SCC of the larynx and hypopharynx, there was no significant difference between IC followed by CRT compared to CRT alone for our primary outcome of FLPS or in our secondary outcomes of OS, PFS, DMFS and laryngectomy rate. On MVA there was also no improvement in outcomes associated with the use of IC. However, the patients in the IC arm were more likely to have clinical stage IV and N2-3 disease, which could have impacted our results. N2-3 disease was associated with worse FLPS, OS, PFS, and DMFS while T stage was not significantly associated with these outcomes. Additional research aimed at identifying which patients undergoing larynx preservation are at highest risk of local failure and are most likely to benefit from IC is needed to further understand the role of IC in the management of patients with locally advanced cancers of the larynx and hypopharynx.
